# A morphometric and stereological study of the chorioallantoic membrane of the developing ostrich (*Struthio camelus australis*) embryo: a light and transmission electron microscopy investigation

**DOI:** 10.1098/rstb.2023.0423

**Published:** 2025-02-27

**Authors:** Dzunani Mthombeni, Andrew N. Makanya, Sikiru Jimoh, John Maina

**Affiliations:** ^1^ Department of Zoology, University of Johannesburg, Auckland Park 2006, Johannesburg, South Africa; ^2^ Department of Veterinary Anatomy & Physiology Riverside Drive, Chiromo Campus, University of Nairobi, Box 30197-00100, Nairobi, Kenya; ^3^ Department of Human Biology, Faculty of Health Sciences, Walter Sisulu University, Mthatha, Eastern Cape, South Africa

**Keywords:** chorioallantoic membrane, development, morphometry, stereology, regression analysis, ostrich

## Abstract

The developing chorioallantoic membrane (CAM) of the ostrich (*Struthio camelus*) was studied between embryonic days 16 (E16) and E37. Egg masses and volumes were estimated prior to harvesting of the CAM. CAM volumes were obtained before the CAM was sampled for histology and transmission electron microscopy analysis. Stereological methods were used to estimate volume densities and absolute volumes of CAM structural components. Growth rate estimates of the CAM and its major components were obtained. At E16, the three layers of the CAM were clearly delineated, but large parts still had not developed the blood–gas barrier (BGB) portions. By E37, chorionic blood capillaries had assumed a superficial position with thin BGB portions covering most of the chorionic surface. On regression analyses, the CAM had two growth phases, namely phase I that occurred between E16 and E25, when the CAM grew rapidly from a volume of 5.55 ± 1.27 to 28.82 ± 5.62 cm^3^ to then decrease to 25.18 ± 4.79 cm^3^ during phase II (E25−E37). The latter decline was attributed to changes in the chorionic and allantoic layers, while regression in the mesoderm mainly characterized blood and lymphatic vessels.

This article is part of the theme issue ‘The biology of the avian respiratory system’.

## 1. Introduction

Enclosing developing embryos and physically intervening between them and the external environment, the chorioallantoic membrane (CAM) or the chorioallantois is a thin, intensely vascularized tissue covering that occurs in eggs of particular amniotes such as birds and reptiles. It develops by fusion of the mesodermal layers of two extra-embryonic membranes, namely the chorion and the allantois [1,2]. The CAM is the avian homologue of the mammalian placenta [3]. It performs multiple vital tasks such as protection, gas exchange and maintenance of acid–base homeostasis, transport of calcium from the eggshell to the developing embryo, elimination and storage of metabolic waste products and reabsorption of water and ions from the allantoic fluid [4]. Cytoarchitecturally, the CAM is a simple, delicate membrane that essentially comprises three parts: (i) the chorion that contacts the inner shell membrane originates from ectoderm; (ii) the allantois that lines the allantoic cavity originates from the endoderm, and (iii) the intermediate mesoderm, which is rich in blood vessels and diverse stromal cells, forms between the two epithelial cell layers [3,5–7].

The structural complexity of the CAM increases with embryonic development, i.e. as the demand for oxygen and nutrients increases. As the embryo grows, the CAM becomes better differentiated into its structural components. As observed earlier by Makanya *et al*. [8], the chick chorion stretches out by recruiting cells from the mesenchymal layer and, after stage E18, the CAM starts to degenerate by apoptosis of the epithelial cells. Interestingly, during incubation, the initially undifferentiated state of the chorionic epithelium may provide a protective function by impeding inflow of ions before the proper time that calcium and bicarbonate ions are required by the developing chick embryo [9].

The avian CAM has proven to be a profoundly instructive research model that has obviated the wasteful and objectionable use of live animals in scientific research in accord with the three R’s (replacement, reduction and refinement) principle [10] that seeks to reconcile animal welfare concerns with animal research. The CAM is a structurally delicate, inexpensive and easily accessible structure that develops fast and therefore gives results quickly. It can be easily experimentally manipulated for *in ovo*, *ex vivo* and *ex ovo* preparations. The CAM has meaningfully been used to study various biological processes and mechanisms such as development, vasculogenesis, angiogenesis, wound healing, xenografting, drug delivery, immune-based studies, cancer research, radiation biology and testing of biomaterials for biomedical tissue engineering (reviewed in [11]).

With the exception of a few species of birds such as the domestic fowl (*Gallus* variant *domesticus*), the mallard duck (*Anas platyrhynchos*), the Japanese quail (*Coturnix japonica*) and the turkey (*Meleagris gallopavo*) [12–14], regrettably, details on the functional design of the CAMs are largely lacking for the eggs of most species of birds, particularly the wild ones. Regarding the suitability of certain CAMs for particular research, applications and methodologies, Kundecová *et al*. [14] observed consequential differences in the biological properties of the CAMs of quail, turkey and duck eggs. The CAMs of the domestic chicken and a few other species of birds have mostly been used for ease of their availability rather than from clear scientific evidence that they are the most suitable ones for certain experiments and investigations [1,5,11,13,15]. For researchers to identify the most instructive CAM for a particular kind of experiment/investigation, there is an urgent need to increase and diversify the number of species of birds on which CAMs have been well-studied.

Recently, a detailed study has documented methods of growing shell-free and in-shell ostrich CAM, with some insights on how it develops [16] but the study lacked important aspects such as growth dynamics or morphometric changes across developmental stages. The ostrich is interesting in being the largest extant bird with the largest CAM.

There are two extant species of the ostrich, namely the common ostrich (*Struthio camelus*) and the critically endangered Somali ostrich (*Struthio molybdophanes*), which was recognized as a distinct species by BirdLife International in 2016 [17]. Native to certain parts of Africa, ostriches are now globally commercially farmed. Flightless, the birds may reach a body mass of 180 kg, lay eggs that are as heavy as 2.6 kg and the strong eggshell may be as much as 3.3 mm thick [18–21]. Scanty data exist on the structure of the CAM of the ostrich egg [18–24] and systematic developmental studies such as those that have been performed on the CAM of the chicken by investigators such as Leeson & Leeson [5], Dunn & Fitzharris [9], DeFouw *et al*. [25], Dimitropoulou *et al*. [26] and Makanya *et al*. [8] are lacking.

The possibility of using positron emission tomography (PET) combined with computed tomography imaging to study *in ovo* developing ostrich embryos was reported by Freesmeyer *et al*. [27]. Here, morphological and morphometric studies of the development of the ostrich CAM at various times of incubation have been performed. The aim was to elucidate the dynamics of the ostrich CAM development and determine whether the structure of the ostrich CAM differs from those of the other species that have been investigated. Furthermore, investigations on the growth rates of the CAM and its components investigated here offer baseline information for future studies involving ostrich CAM.

## 2. Material and methods

### (a) Experimental animals

This study was approved by the Animal Ethics and Control Committee of the University of Johannesburg (permit number RP 0785211). Fertilized ostrich eggs from South African black ostrich (*Struthio camelus australis*) were obtained from Klein Karoo International Company of the Oudtshoorn area of the Little Karoo Province of South Africa. The eggs were carefully packed in paper cartons and cushioned with shredded paper to minimize shock and breakages and transported to the Department of Zoology, University of Johannesburg, where further incubation and experimentation was conducted. The number of eggs and the procedures applied are shown in table 1,1.

**Table 1.** Number of animals used per technique and age group. BGB, blood–gas barrier; CAM, chorioallantoic membrane; LM, light microscopy; TEM, transmission electron microscopy.

technique	age	number of animals	remark
LM	E16	4	specimens used for estimation of CAM volume and morphometry of the CAM components
	E25	5	
	E36	5	
	E37	5	
TEM	E16	3	specimens used for the study of blood–gas barrier development and measurement of the arithmetic mean thickness of the BGB
	E25	3	
	E36	3	
	E37	3	

### (b) Incubation of eggs to obtain chorioallantoic membrane

To study the CAM, eggs were incubated in an Inco Therm incubator maintained at 36.5°C and 65% humidity.

### (c) Egg volume and mass

The egg volumes and masses were determined using direct methods as described by Alikhanov *et al*. [28]. Briefly, they were placed on an electronic weighing balance and the mass was recorded. Then, the egg was placed in a large calibrated beaker half-filled with water. Its volume was recorded after immersing it gently in the beaker.

### (d) Windowing of eggs

Windowing of eggs allows access to the CAM while the embryo remains in shell. To capture the developing CAM, eggs were windowed at selected time points. Windowing was done on the broad side with the air cell (top windowing). Air cell identification was done by candling [29]. The Tork Craft Mini Rotary Drill fitted with a fine bit was used as detailed elsewhere [16]. At different days of incubation, embryonated eggs were opened and the embryo together with all the membranes and their contents carefully separated from the shell but maintained within the shell membrane. Embryos at 26 days of incubation or older were anaesthetized by intraperitoneal injection of sodium pentobarbitone. The CAM was carefully separated from the embryo and yolk sac and washed in phosphate-buffered saline, immersed in a solution of 2.5% glutaraldehyde in 0.1 M cacodylate buffer (pH 7.4, 350 mOsmol kg^−1^ H_2_O). The CAMs were kept in the fixative for at least 24 h before further processing was done.

The embryo mass was determined, and after at least 24 h of fixation, the CAMs were examined under a dissection microscope and any parts of the shell membranes, albumin or yolk were carefully removed with fine plastic forceps. The CAMs were stored in the same fixative awaiting further processing. The total volume of CAM was determined by the Scherle’s weight displacement method [30].

### (e) Chorioallantoic membrane sampling for morphometry and stereology

Fixed CAMs were placed on wax plates and diced into quadrats measuring approximately 5 cm × 5 cm. Samples to be processed further were picked by systematic random sampling [31]. The selected slice was divided into two halves, one of which was processed for light microscopy (LM, paraffin embedding) while the other one was processed for transmission electron microscopy (TEM).

### (f) Light and transmission electron microscopy

For morphometric analysis, specimens were dehydrated through ascending concentrations of ethanol, embedded in paraffin wax and sections obtained at a nominal thickness of 5 μm. For semithin and TEM sections, CAMs were cut into smaller slices and postfixed in osmium tetroxide, block-stained using uranyl acetate, dehydrated through ascending concentrations of ethanol and embedded in epoxy resin. Semithin sections were obtained at a nominal thickness of 1 μm, stained with toluidine blue and viewed under a digital light microscope. Ultrathin sections were obtained at a thickness of 90 nm, counterstained with lead citrate and viewed on a transmission electron microscope.

### (g) Morphometric estimations

A quadratic lattice grid etched on a graticule was superimposed on the paraffin sections. The intersections between the grid lines formed the points that were used to analyse various components of the CAM. The number of points falling onto the structures of interest was counted. Volume densities of the various structural components were determined as the ratio of the number of points falling onto the component of interest to that of the reference space. For example, the formula used for the volume density calculations for the chorion across all incubation days was as follows:


$$Vvc=\frac{Pc}{Pt};$$

where *P*c represents the number of points falling onto the chorion and *P*t is the total number of points in a particular field. The same formula was applied for the determination of volume densities for all the parameters across all the incubation days. The volume densities were expressed as percentages.

Then, *V*c, the volume of the component of interest, was estimated from the formula:


Vc=Vvc∗V(ref);

where *V*(ref) is the volume of the reference space.

### (h) Data analysis

The mean volume densities and mean BGB thickness measurements along with the standard deviations of the different structural parameters were calculated. These values were used to perform statistical analyses to determine differences among the volumes of CAM as well as the proportions of the various CAM components and their absolute volumes at selected incubation days. One-way ANOVA was used to detect differences among the various study groups. One-way ANOVA was used when targeting single independent variables (e.g. incubation age) to investigate if variations in the latter factor had measurable effects on a dependent variable (e.g. egg mass, egg volume, CAM volume, etc.). Tukey’s test was used to perform *post hoc* analysis of the targeted values using Social Science Statistics Software free online at: https://www.socscistatistics.com/tests/anova/default2.aspx. The level of significance was set at less than 5% (*p* < 0.05).

To determine trends in the various changing parameters, least squares regression analysis was performed and the coefficient of variation (*r*) estimated to determine the goodness of fit. All non-ratio data were log-transformed and the power equation,


$$log_{10}Y=log_{10}a+blog_{10}X$$

was employed to calculate the scaling exponent (*b*), and hence, the relevant allometric equation was obtained. Based on the growth dynamics of the CAM obtained from regression analyses, the growth pattern was divided into phases.

## 3. Results

### (a) Structure of the chorioallantoic membrane and the development of the blood–gas barrier

**Figure 1.**
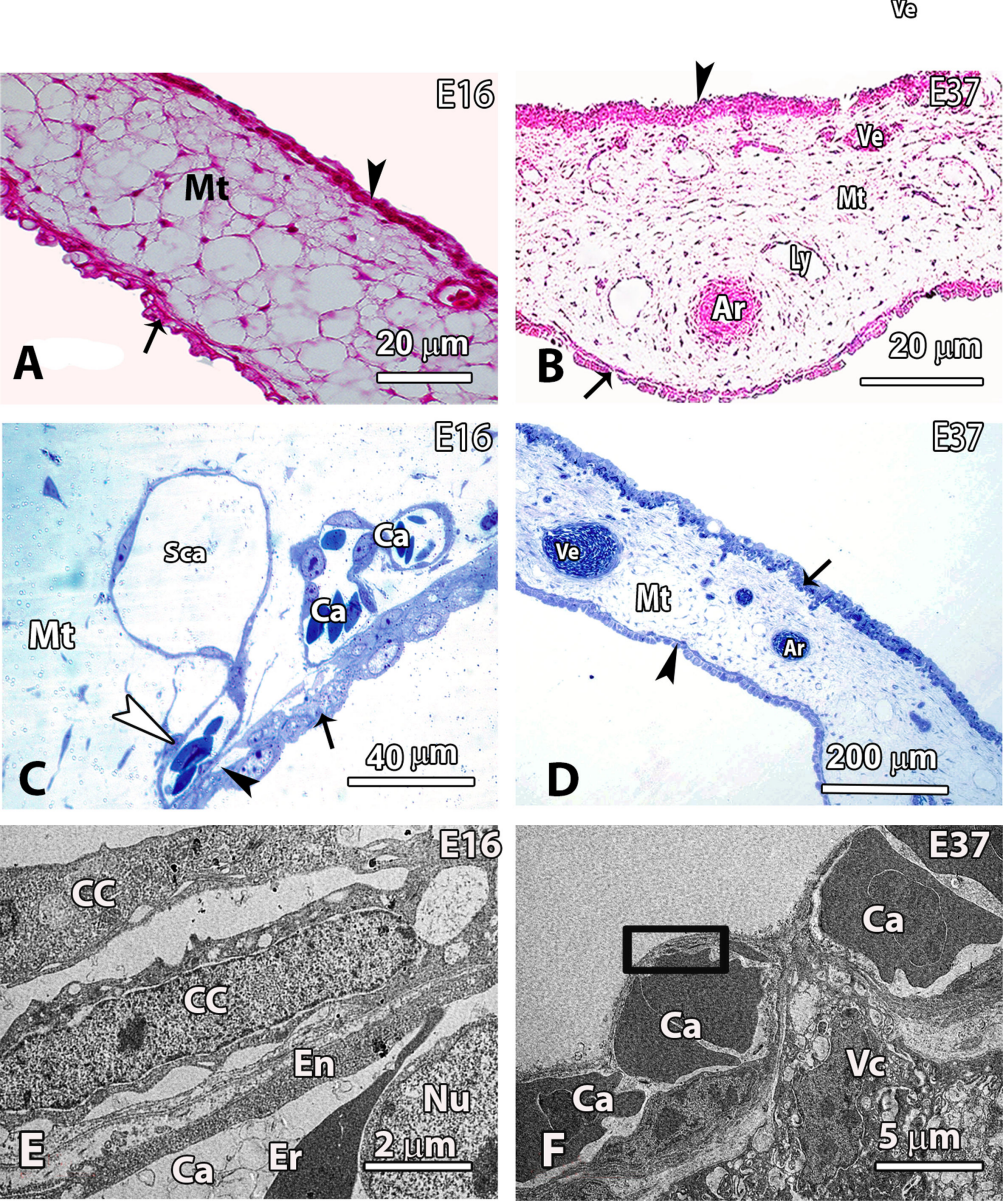
Histological, semithin and thin sections showing the structure of CAM at various developmental stages. (A,B) Histological sections showing the structure of CAM at various developmental stages. The sections were used to estimate the volume densities of the various components of the CAM and hence the absolute volumes. At E16 (*a*), the CAM comprises abundant mesenchyme (Mt) sandwiched between the developing chorion (arrowhead) and the allantois (arrow). By E37 (*b*), the components of the mesoderm are well differentiated and the arteries (Ar), veins (Ve) and lymphatics (Ly) are discernible surrounded by mesenchyme. At this stage, the CAM is mature and both the chorion (arrowhead) and the allantois (arrow) are clearly defined. (C,D) Semithin sections showing the development of the BGB as well as the layers of the CAM at various developmental stages. At E16 (*c*), the Mt is abundant and the chorion comprises largely a low cuboidal epithelium (black arrowhead) on which exchange capillaries (white arrowhead) abut to form the BGB portions. Large portions of the epithelium are devoid of capillaries (arrow) while some large sinusoidal capillaries (Sca) and capillaries approaching the epithelium (Ca) are encountered in the mesodermal layer. At E37 (*d*), the CAM is clearly delineated into a mature chorion (black arrow), an allantois (black arrowhead) and a mesoderm with arteries, veins and intervening mesenchymal tissue. (E,F) TEM sections showing the development of the BGB at various stages. The sections were used to estimate the arithmetic mean thickness of the BGB. At E16 (*e*), the capillary covering cells (CC) of the chorion sometimes occurred in two layers next to a prospective gas exchange capillary. The endothelial cell nucleus (Nu) is located on the internal aspect of the capillary to allow the establishment of a thin BGB. The erythrocytes (Er) in the capillary lumina are also shown. By E37 (*f*), a very thin BGB (rectangle) measuring less than 1 µm in thickness is established. Notice the nucleus of the endothelium (En) on the internal aspect of the exchange capillary and a villous cavity cell (Vc) between the capillaries. Ar, arteries; Ca, capillaries; CC, capillary covering; En, endothelium; Er, erythrocytes; Ly, lymphatics; Mt, mesenchyme; Sca, sinusoidal capillaries; Vc, villous cavity; Ve, veins.

The main components of the CAM that were investigated in the current study included the chorion, the mesoderm and the allantois. The major components of the mesoderm, namely the mesenchyme, the arteries, the veins and lymphatics were also studied. The latter vessels were identified as those that were ≥25 μm in diameter. The growth of the CAM and its components was followed from E16, through to E37 using LM thin paraffin sections, semithin sections and ultrathin sections using TEM (figure 1).

At E16, the CAM comprised abundant mesenchyme sandwiched between the developing chorion and the allantois. By E25, the components of the mesoderm were well differentiated and the arteries, veins and lymphatics were discernible surrounded by mesenchyme (data not shown). At E37, the CAM was mature and both the chorion and the allantois looked much thinner (figure 1).

A closer investigation of semithin sections (figure 1) revealed the stages of development and distribution of the BGB portions. At E16, blood capillaries in the mesoderm approximated the epithelium lining the air space to form the BGB portions. Large parts of the epithelium lacked blood capillaries while some large sinusoidal capillaries grew toward the epithelium to form the thin parts of the BGB (figure 1C,D). At E37, the CAM was clearly delineated into a mature chorion, an allantois and a mesoderm with arteries, veins and lymphatics in the intervening mesenchymal tissue (figure 1).

At the ultrastructural level, the dynamics of the formation of the BGB could be followed (figure 1). At E16, the prospective capillary covering (CC) cells of the chorion in some cases occurred in two layers next to a developing gas exchange capillary. Occasionally, very thin parts of the BGB were observed: they formed the sites of gas exchange in the developing CAM. The endothelial cell nuclei of the embryonic blood capillaries were deeply located, i.e. they were situated on the internal aspect of the capillary endothelium to allow formation of a thin BGB. By E37, a very thin BGB that measured less than 1 µm in thickness was established (figure 1).

### (b) Blood–gas barrier thickness

**Figure 2.**
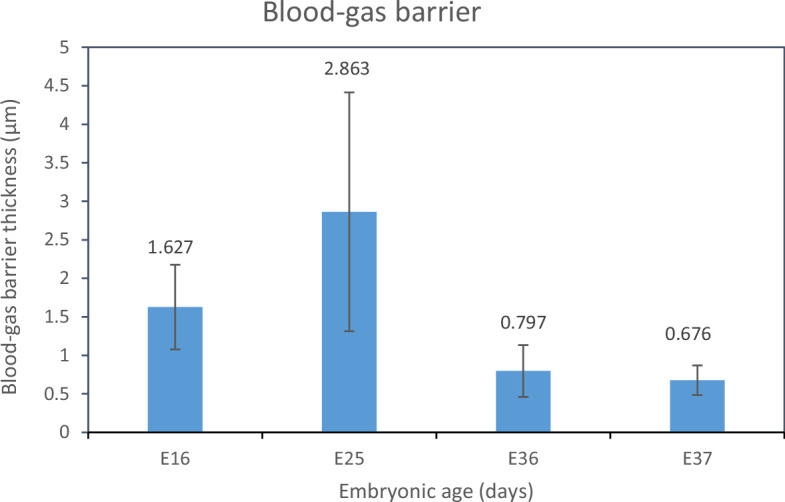
Arithmetic mean thickness of the blood–gas barrier of the CAM at various incubation ages. The value was high at E16 (1.627 µm) and increased to a peak at E25 (2.863 µm) after which it decreases to less than 1 µm by E36.

Estimation of the arithmetic mean thickness of the BGB of the CAM at various incubation ages revealed that the value was high at E16 (1.627 ± 0.55 µm), increased to a peak at E25 (2.863 ± 1.55 µm) then decreased to less than 1 µm (figure 2).

### (c) Egg mass and volume during incubation

**Figure 3.**
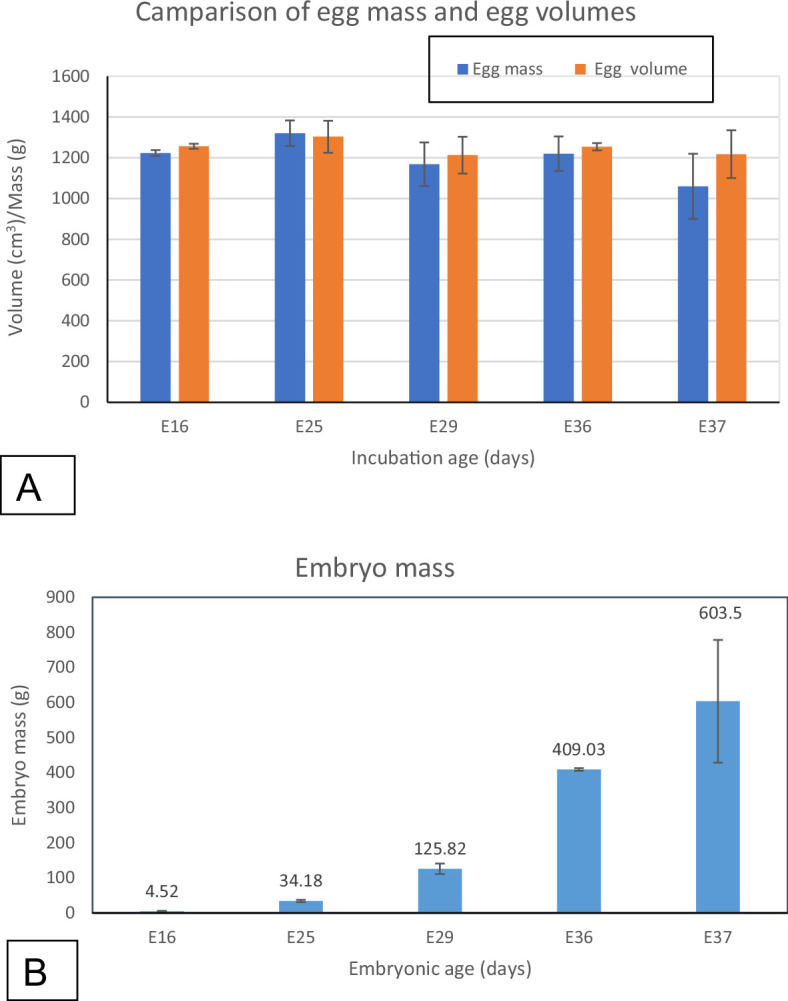
Bar graphs showing egg weight and egg volume at various incubation ages (A) and the variation in body mass (B). (*a*) Egg volume does not differ among the various incubation ages but egg weight appears to decrease slightly towards hatching. (*b*) The embryo body mass increased from 4.52 ± 1.63 g at E16 to 603.5 ± 174.71 g at E37.

As expected, egg mass and egg volume did not appear to vary significantly during incubation (one-way ANOVA) although at E37, there was a slight reduction in egg mass. Using Tukey’s *post hoc* analysis test, it was shown that there were no significant differences between the egg masses and the egg volumes for the various incubation days (figure 3).

### (d) Embryo mass and chorioallantoic membrane volume

**Figure 4.**
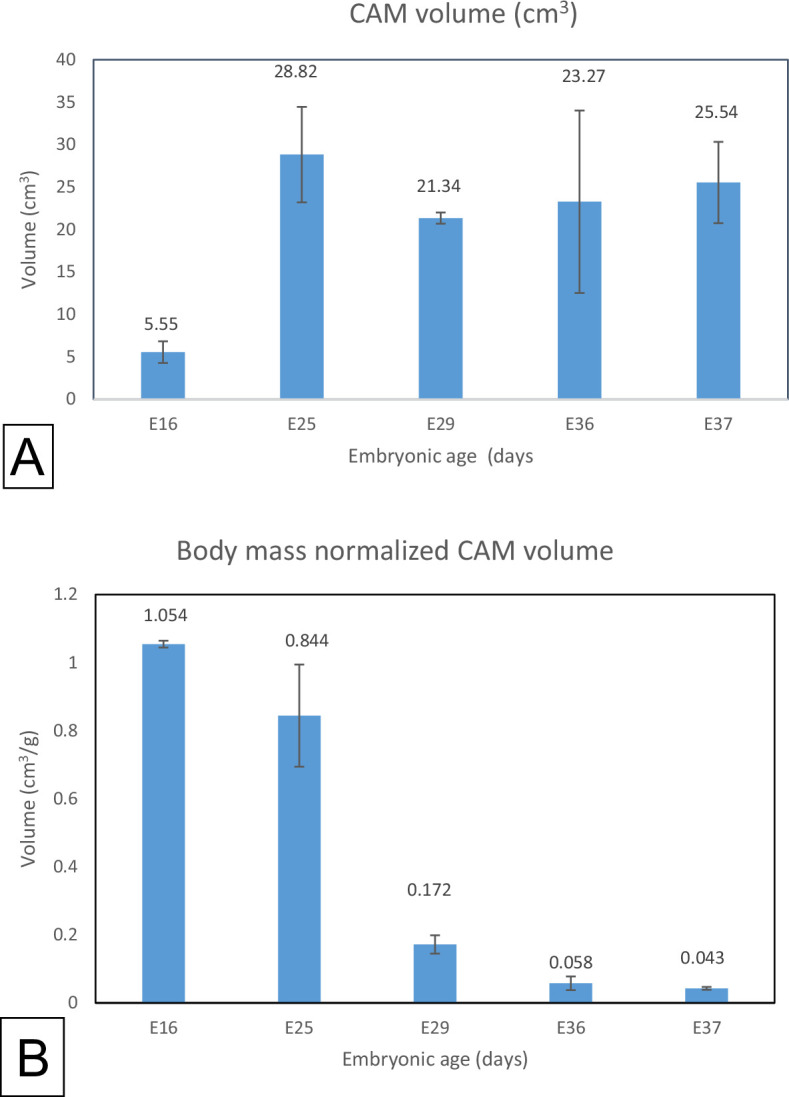
Bar graphs showing the variation in the CAM volume (cm^3^) and body mass normalized CAM volume (cm^3^ g^−1^). (A) The CAM volume increased sharply from E16 to a maximum at E25, decreasing steadily in phase II of development. (B) As expected, body mass-normalized CAM volume decreased steadily towards hatching.

In phase I, the embryo mass increased from 4.52 ± 1.63 g at E16 to 409.03 ± 3.89 g at E25, a growth of 656%. The percentage increase in phase II was a remarkable 1680%, i.e. from 409.03 ± 3.89 g at E16 to 603.5 ± 3.89 g at E37 (figure 4; table 2). In contrast, CAM volume increased most rapidly in phase I (419%) while in phase II, the volume decreased (−11%).

**Table 2.** Growth phases and percentage changes in body mass and CAM volume in the developing ostrich embryo.

phase	age ranges	body mass range (g)	increase (g)	% increase
phase I	E16–E25	4.52–34.18	29.66	656.2
phase II	E25–E37	34.18–608.5	574.32	1680.3

The CAM volume reached a maximum value at the end of phase I and then decreased in phase II. The CAM volume increased sharply from E16 to a maximum at E25 (table 2, figure 4A), decreasing steadily in phase II of development. As expected, body mass-normalized CAM volume decreased steadily towards hatching (figure 4B).

### (e) Volume densities of the main structural components of the chorioallantoic membrane

The volume densities of the mesoderm, the chorion and the allantois are presented in electronic supplementary material, figure S1. Interestingly, and as expected, the mesoderm took the greatest percentage throughout with its contribution being lowest at E16 (77.06%), increasing to 81.05% by E25. The proportion did not change much (only 84.55% at E36) but this greatly increased to 91.69% at E37. The percentages of the chorion remained slightly higher than those of the allantois but both parameters decreased steadily towards E37. The volume density of the mesodermal layer was significantly greater than that of the chorion and the allantois at all embryonic ages. Although the volume of the chorion remained consistently higher than that of the allantois throughout the embryonic ages, the difference was not statistically significant.

### (f) Regression analysis

**Figure 5.**
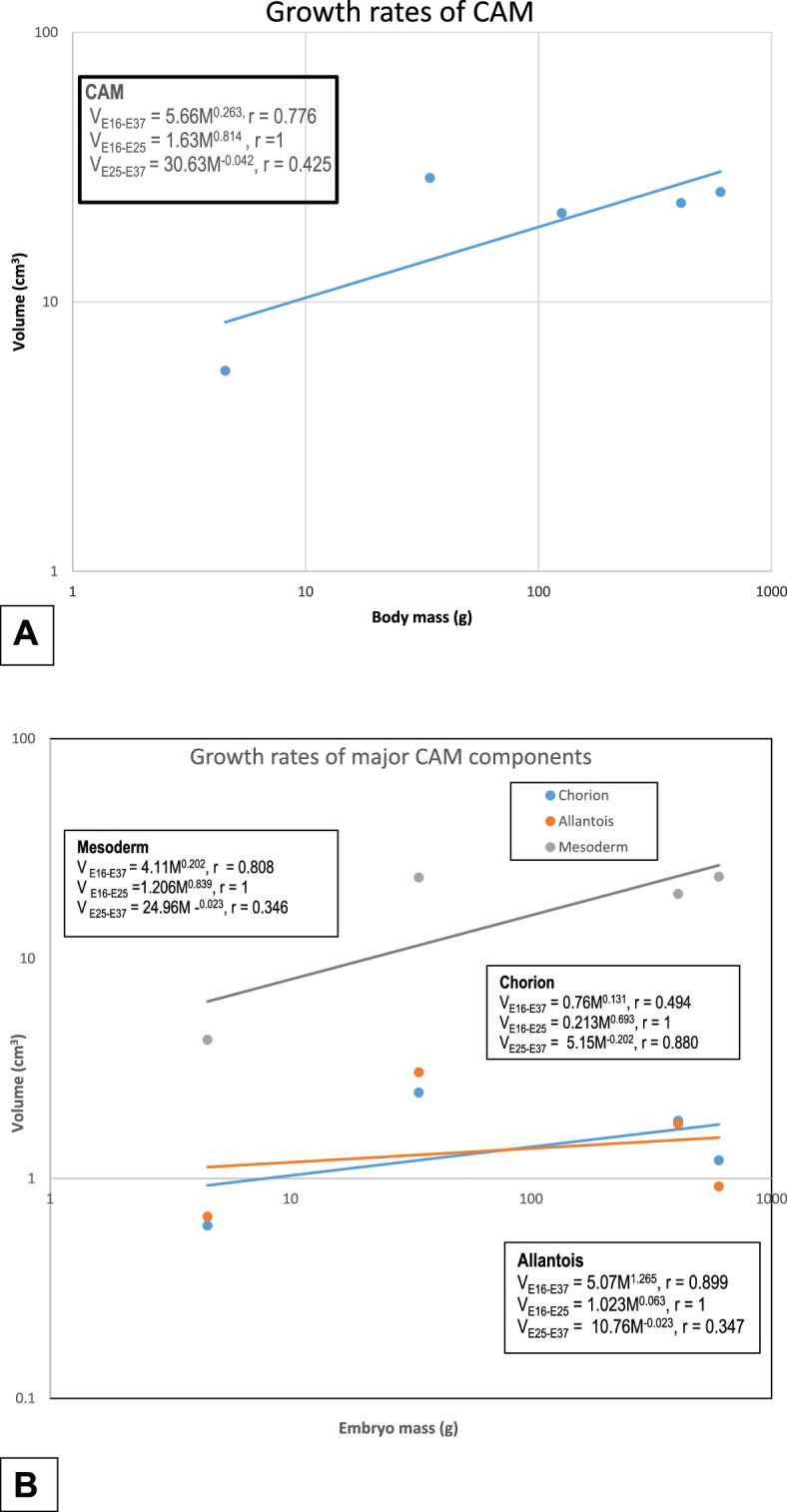
Logarithmic line graphs showing growth rates of CAM (A) and the various coarse components of the CAM (B). Notably, the CAM and CAM components had two phases of growth. Phase I (E16–E25) was characterized by robust growth of all, while phase II had moderate growth coupled with some degree of regression for some components.

The CAM was growing fastest in phase I where a strong correlation with body mass existed (figure 5). In phase II, the CAM was regressing and the volume was only moderately correlated with body mass. The mesoderm was the fastest growing component in phase I. Both the chorion and the allantois grew at the same rate. At various growth phases, when considered on an individual basis, all components were growing fastest in phase I and except for the mesoderm, they were regressing in phase II. All the structural components significantly correlated to body mass during the entire period (figure 5).

Among the mesodermal components, growth of the mesenchymal tissue, the arteries and lymphatics were all strongly positively correlated with the body mass in phase I but were regressing in phase II. Growth of the veins weakly correlated with body mass but this changed to a strong correlation in phase II (electronic supplementary material, figure S2).

The mesenchymal tissue grew rapidly in phase I but declined slightly in phase II. The arteries displayed a similar trend but the reduction in their volume density was more notable than that of the mesenchyme (electronic supplementary material, figure S2). Both the veins and the lymphatics grew rapidly in phase I but regressed in phase II (electronic supplementary material, figure S2). All the structural components that were analysed significantly correlated with body mass during the entire period of embryonic development.

## 4. Discussion

Comprising reptilian, mammalian and avian tetrapod vertebrates, the Amniota clade is characterized by, among other attributes, eggs of which embryos develop surrounded by a set of three extraembryonic membranes, namely the amnion, the chorion and the allantois [32–35]. The cleidoic (self-supporting = contained = shielded) shelled amniotic egg [36] that conserves water under desiccating environmental conditions constitutes a fundamental reproductive adaptation which permitted transition from life in water onto terra firma [34,37,38]. Birds are the most speciose and widely dispersed extant air-breathing vertebrates that comprise approximately 11 000 species [39,40]. Interestingly, although notable mortalities occur during egg development, from laying (oviposition) and infection of the reproductive system [41], for still unclear reasons, birds have remained exclusively oviparous [42–45].

In birds, the laying of eggs is a complex process that is driven by environmental conditions, hormonal effects and genetic factors [46–49]. In some instances, abnormalities of eggs and complications during laying occur. With few exceptions of groups of birds such as the ‘incubator birds’ (megapodes) and ‘brood parasites’, e.g. the cuckoos, the eggs are fertilized internally, laid and directly incubated: the embryos develop inside the egg and chicks emerge (hatch) at the end of the incubation period.

During incubation, it is known that water loss from eggs occurs to create space for air in the air cell [36,46,47]. In this study, however, there was no significant egg mass change in the embryonated eggs; only a slight decrease in egg mass was observed at E37. Notably, the last embryonic age studied here was 5 days short of the expected hatching day.

As described recently [16], the ostrich CAM takes about twice as much time to develop as that of the chicken but the events that take place are apparently comparable. The time points chosen in the current study correspond roughly to important times in the development of the extensively studied chicken CAM. In the chicken CAM, fusion of the chorion and allantois is complete by E12 [5], and in the ostrich, this is accomplished by E25. Indeed, this is the embryonic age at which the highest CAM volume is attained and the arithmetic mean thickness of the BGB is greatest. The formation of the tripartite CAM is mediated partly by fusion of an epithelial-shaped mesothelial layer of the chorion with that of the mesoderm, which breaks up after fusion, presumably by undergoing epithelial–mesenchymal transition [48]. Further growth entails remodelling and shifting of both the nuclei of CC cells to regions between adjacent blood capillaries as well as movement of endothelial cell nuclei to the inner aspect of the endothelium. The latter events ensure that a thin BGB of less than 1 µm thickness is attained by E36.

The dynamics of formation of the BGB in the CAM have received very little attention from researchers. In the chicken CAM, a harmonic mean thickness of the BGB of 0.47 µm was reported [49] but there are no data on the arithmetic mean thickness. An arithmetic mean thickness of the BGB of 0.797 µm was determined in this study for the ostrich CAM at E36. Showing the possibility that this value would reduce towards hatching, the arithmetic mean thickness of the ostrich CAM at E37 was 0.676 μm.

Details of the structure, the development and the remodelling of the vertebrate BGB have been well documented [50]. In the mammalian lung, this proceeds through conversion of type II cells to type I cells: thinning and elongation of the cells as well as extrusion of the lamellar bodies (reviewed in [51]). In the non-compliant avian lung, such processes are more complex and less well understood [52–54]. The processes are termed peremerecytosis if they involve cell squeezing and constriction, or secarecytosis if they entail cutting cells to size. These processes ultimately result in a thin BGB that promotes passive diffusion of gases [53].

Although cell-cutting processes have been described in the ostrich lung [54], here, no such events were encountered in the formation of the BGB in the CAM. Cells of the early chorionic epithelium, which were initially low cuboidal became adherent to incipient blood capillaries in the periphery of the mesoderm. The cells subsequently had their nuclei shift to the periphery, thinned out and, as they became apposed to the capillary endothelium, they slotted in a thin layer of the extracellular matrix. The latter formed a common basement membrane. Details and dynamics of the formation of the CAM BGB, however, require further investigations.

Notably, for the times studied here, the ostrich CAM displayed two growth phases compared to the three described in chicken CAM [8]. From E16 to E25, generally, there was rapid growth of the CAM, with an increase of its volume by 419%, a time when the maximum volume was attained. In contrast, the growth of the embryo was delayed a bit in phase I but in phase II it increased by 1680%. This is plausible since the CAM needs to develop first so that it can supply the developing embryo with the requisite nutrients and gases.

As seen in the decrease in their volume densities, regression of the CAM mainly involved the chorion and allantois while the mesoderm declined slightly and then increased. In previous studies, apoptosis was reported both in the chorion and the allantois [8]. Future studies on ostrich CAM growth should focus on the events occurring very early during incubation and closest to hatching.

## Data Availability

All data are available as supplementary material [55].
